# The effect of metal mixture composition on toxicity to *C*. *elegans* at individual and population levels

**DOI:** 10.1371/journal.pone.0218929

**Published:** 2019-06-25

**Authors:** Sofie Moyson, Raewyn M. Town, Kris Vissenberg, Ronny Blust

**Affiliations:** 1 Systemic Physiological and Ecotoxicological Research (SPHERE), Department of Biology, University of Antwerp, Antwerp, Belgium; 2 Integrated Molecular Plant Physiology Research, Department of Biology, University of Antwerp, Antwerp, Belgium; 3 Plant Biochemistry & Biotechnology Laboratory, University of Applied Sciences Crete – Technological Educational Institute, Department of Agriculture, School of Agriculture, Food & Nutrition, Stavromenos, Heraklion, Crete, Greece; CINVESTAV-IPN, MEXICO

## Abstract

The toxicity of zinc (Zn), copper (Cu), and cadmium (Cd) to the nematode *Caenorhabditis elegans* was characterised under single metal and mixture scenarios at different organisational levels. The effects on population size and body length were investigated at two concentrations corresponding to the 24 h LC5 and LC20 levels. Metal toxicity was dependent on metal concentration, exposure time and mixture composition. Populations exposed to LC20 levels of Cd, ZnCu, CuCd and ZnCuCd plummeted, while for all LC5 concentrations, population size continued to increase, albeit that single metals were less harmful than mixtures. Combinations of the LC20 concentration of Cd with a range of Zn concentrations showed concentration dependent mitigating effects on population size and antagonistic effects on mortality. By combining effects at different organisational levels, more insight into metal toxicity was obtained. Metal effects were more evident on population size than on body length or mortality, suggesting that population size could be considered as a sensitive endpoint. Furthermore, our observations of ZnCd mixture effects at the individual and population levels are consistent with literature data on the dose-dependent expression of the *cdf*-2 gene, which is involved in mediation of Zn and Cd toxicity.

## Introduction

Metals of natural and anthropogenic origin can be mobilised in environmental compartments and thereby pose a toxicological risk. Essential metals (e.g. Zn, Cu) are needed for biological functions, while non-essential metals (e.g. Cd, Pb) may disrupt biological processes depending on their concentration, dynamic chemical speciation, bioavailability and biological species sensitivities. Exposure to nonessential metals and to a deficiency or an overload of essential metals can be detrimental to many animals. For ethical reasons, toxicological studies are preferably performed on non-vertebrate organisms, e.g. soil nematodes such as *C*. *elegans*.

*C*. *elegans* is a bacterivorous nematode that is ubiquitous in soil and fulfils an important ecological role. It is a well characterised model organism that has found wide application in mechanistic studies on the toxicity of metals, organic compounds, and nanoparticles [1–5]. The life cycle involves rapid development over 3–4 days through four larval stages (L1-L4), production of oocytes by the adult hermaphrodite during a 4-day fertile period, then the mature adults live for a further 10–15 days. When the environmental quality is not optimal, a developmentally arrested third larval stage, dauer larva, is formed [6]. Adult lifespan, lifetime fecundity and body length vary according to environmental quality [6]. Body length increases with life stage, from ca. 370 μm at first molt, to 1060 μm when egg laying starts (T = 20°C) [7]. Adult *C*. *elegans* typically range from 1–1.5 mm in length [8]. The highly predictable developmental features of *C*. *elegans* cause their growth responses to be good toxicological endpoints.

Due to the above mentioned life history traits and known genome, *C*. *elegans* has been used to study the toxic effects of single metals on various endpoints ranging from the molecular to the population level. For example, at the molecular level, exposure to metal ions induces expression of genes and induction of proteins involved in maintaining ion homeostasis and regulating toxicity [9–12]. Whilst at the individual and population level, a reduction of bioluminescence, movement, feeding, growth and reproduction, a delay in egg laying and an increased generation time were reported after exposure to Zn, Cu and/or Cd concentrations in the LC20-LC50 range [8,13–18]. However, there is a paucity of studies that consider the combined toxic effects at different organisational levels. Furthermore, in contrast to single metals, knowledge about metal mixture effects is still scarce. Metal mixture toxicity is typically evaluated in terms of the “concentration addition” (CA) model or the “independent action” (IA) model. In both cases, deviations from the expected toxicity are interpreted as being the result of interactions between the metals. The extent of deviation deemed to be significant, can be based on a statistical approach or simple judgement [19]. Interactive effects of metals can be additive, antagonistic or synergistic. The potential interplay between the biotic handling processes for different metal ions means that the nature and timescale of toxicity responses under mixture conditions does not follow straightforwardly from observations on single metal exposures. Testing mixtures and the corresponding individual metals simultaneously is thus necessary to obtain insight into the combined actions. Most studies on metal toxicity to *C*. *elegans* correspond to short exposure periods (24 h or 48 h) [15,20–22].

In our previous study the effects of single metals and mixtures on the mortality and behaviour (locomotion and chemosensation) of *C*. *elegans* after short exposure times, i.e. 24 h and 48 h, were described [23]. However, there is a paucity of information on the longer term effects of metals, even for single metal exposures. It is known that the adverse effects of Cd become more apparent after a few days of exposure [24], which may influence its contribution to the toxicity of mixtures as a function of time. Furthermore, toxicity to individuals can result in e.g. mortality and decreased development, which will be reflected at the population level in lower abundance, altered distribution of the life stages present in the population etc. Population size, i.e. the total number of live individuals at a given time point, can thus be considered as a holistic endpoint that integrates individual life histories and trans-generational effects. *C*. *elegans* is able to alter both its larval development and its reproductive strategy (e.g. lifetime fecundity and time of reproduction) in response to environmental stress [6] and the influence of pollutants on the population growth of *C*. *elegans* has been proposed as a means to assess long-term environmental effects [1,25].

Herein we assess the utility of population size as a potential sensitive endpoint for metal toxicity. The aim of the present study is to gain insights into the sensitivity of *C*. *elegans* to selected metals (Cu, Cd, Zn) and to investigate whether and how these sensitivities are mutually affected in mixture exposure scenarios at the individual (body length) and population level (population size), and the time dependence thereof (10–12 days). To better understand the mitigating effect of Zn on Cd toxicity, mortality and population size of nematodes were studied after 24 h and 48 h of exposure to different LC concentrations of Zn, whether or not in combination with LC20 of Cd. The main objectives of our study were to identify toxic effects at the individual and population level following exposures to Cu, Cd, and Zn separately and in various mixture combinations, as a function of concentration and exposure time.

## Materials and methods

### *Caenorhabditis elegans* culture

*Caenorhabditis elegans* nematodes, wild type N2 strain, were obtained from the Caenorhabditis Genetic Centre, Minneapolis, USA. NGM agar plates, seeded with *Escherichia coli* (OP50 strain) as food source, were used for nematode maintenance at 20°C [26]. To ensure that nematodes of the same age were used at the start of all experiments, bleaching was performed by adding a hypochlorite solution (5 N NaOH, 8% sodium hypochlorite) to mixed-stage *C*. *elegans*, thereby killing the nematodes that were not protected by an egg shell. Eggs were then raised on NGM plates without food to arrest their development for 24 h. In this way, highly synchronous L1 larvae were obtained. The synchronous L1 nematodes were transferred to OP50 seeded NGM plates; 60 h later, all nematodes were in the adult stage, which was the start point for all experiments.

### Test media

Two concentrations of metals were studied in single and mixture scenarios. We chose concentrations corresponding to the 24 h LC5 and LC20 levels (Table 1), which were determined previously [23] for the adult life stage. For convenience, we subsequently use the terminology “LC5” and “LC20” to denote these concentrations, even though herein we studied effects at times beyond 24 h. Exposure media were prepared from CdCl_2_.2.5H_2_O (Alfa Aesar), CuCl_2_.2H_2_O (Merck A.G.) and ZnCl_2_ (Alfa Aesar) in K-medium, supplemented with *E*. *coli* bacteria (1.5–1.7 g wet weight / L). To prepare the *E*. *coli*, falcon tubes containing 10 mL of LB agar inoculated with OP-50 were incubated for 24 h at 37°C in a shaking incubator at 200 rpm. Subsequently, the solution was centrifuged at 2000 g for 20 min to obtain a bacteria pellet. This pellet was subsequently rinsed with K-medium for three cycles and centrifuged for 5 min at 2000 g to yield a clean pellet. The clean bacteria pellet was then suspended in the test solution at a concentration of 1.5–1.7g wet weight / L. The metal concentrations used are in line with reported metal contents of polluted soils that have been used for toxicity studies with *C*. *elegans* [27]. The various mixtures ZnCu, ZnCd, ZnCuCd, and CuCd were prepared by combining the corresponding LC5 or LC20 concentrations of the individual metals. Metal concentrations were verified by ICP-OES (ICAP 6300 Duo, Thermo scientific) (95%–115% recovery). Since exposure media contained *E*. *coli* bacteria, samples were first freeze-dried (Heto Powerdry LL 30000, Thermo Scientific), 250 μL concentrated nitric acid (TraceMetal Grade, Fisher Chemical) was added and all samples were digested at 110°C for 30 minutes using a heating plate (HotBlock, Environmental Express). MilliQ water was added to make the total volume up to 10 mL.

**Table 1:** LC values of Zn, Cu and Cd for adult *C*. *elegans* after 24 h of exposure.

	LC2		LC5		LC20		LC40		LC60	
	(mg/L)	(mM)	(mg/L)	(mM)	(mg/L)	(mM)	(mg/L)	(mM)	(mg/L)	(mM)
**Zn**	0.445 ± 0.314	0.007 ± 0.005	1.416 ± 0.771	0.022 ± 0.012	9.501 ± 2.841	0.145 ± 0.043	31.495 ± 6.186	0.482 ± 0.095	84.823 ± 17.601	1.297 ± 0.269
**Cu**	NA^a^	NA	0.226 ± 0.104	0.004 ± 0.002	1.299 ± 0.409	0.020 ± 0.006	NA	NA	NA	NA
**Cd**	NA	NA	0.474 ± 0.289	0.004 ± 0.003	7.110 ± 2.315	0.063 ± 0.021	NA	NA	NA	NA

^a^ NA = not applicable

To allow metal partitioning between the aqueous phase and the bacteria, prior to toxicity testing, metal solutions were first incubated with the bacterial suspensions for 12 h (at 4°C to suppress bacterial growth). For each sample of each experiment, the determined pH before (5.1 ± 0.1), during and at the end of the experiment of 48 h (5.4 ± 0.3), 10 days (6.0 ± 0.3) and 12 days (7.2 ± 0.3) was within an acceptable pH range for *C*. *elegans*, thus excluding any potential effects of pH variation on the measured parameters. For each treatment three independent replicates were made.

### Determination of population size and body length

24 h after bleaching, the synchronous L1 nematodes were transferred to OP50 seeded NGM plates. After 60 h, approximately 10 adult nematodes were transferred to a 24-well plate filled with 1 mL K-medium (52 mM NaCl, 32 mM KCl, 5 μg/mL cholesterol, pH 5.1) [28] or test medium (K-medium containing the test metal concentration(s)), supplemented with *E*. *coli* OP50 (1.5–1.7 g/L). The well plates were continuously shaken (160 rpm, 20°C). For all 8 treatments (i.e. control, Zn, Cu, Cd, ZnCu, ZnCd, CuCd and ZnCuCd), the population size (i.e. number of live larvae and adult nematodes) was determined over a period of 10 days when exposed to LC20 levels of metal ions and over 12 days when exposed to LC5 levels. During a long exposure period, multiple generations can be formed and thus the total population size provides insight into trans-generational effects. Every 36 h, medium with nematodes was transferred to a new well with fresh medium (metal stock with *E*. *coli*) (both in equal volumes) and was split in two, thereby reducing the number of nematodes by a factor of 64 at day 10 [6,29]. However, the number of nematodes in groups exposed to LC5 metal concentrations was exceptionally reduced by a factor of 4 at day 6 and the control, Zn and Cu group were reduced by a factor of 8 at that time. To be able to count the number of nematodes, the number in the control LC20 group was further reduced starting from day 6 onwards: 64x at day 6, 128x at 8 day, 512x at day 9, 2048x at day 10. Details of the dilution protocols are given in S1 Table. Our protocol ensured that nutritional factors were sufficient throughout the exposures, i.e. changes in population size cannot be ascribed to a deficiency of bacteria. Every 24 h, the number of living nematodes was counted in each well by taking a picture and making a video of 10 seconds (15 fps) with a camera (Nikon DS-Ri1), attached to a stereomicroscope (Nikon AZ100). Nematodes that were not moving or did not respond to gentle plate shaking, were considered to be dead. These videos were also used for body length measurements. Body length differences between generations were very clear, enabling larvae from one generation to be easily distinguished from the larger subsequent life stages of the previous generation (S1 Fig). The body length of the post-larval stage nematodes (not necessarily from the same life stage) was measured after 0, 5 (approximate time at which a second new generation can be formed) and 10 days of exposure and also after 12 days of LC5 exposure, using Image-J software. (https://imagej.nih.gov/ij). At each time point, 20 nematodes were counted in each of the three replicates. In cases where a second generation developed, the measurements at 10 days of exposure will represent the average size of post-larval nematodes from mixed generations. Nevertheless, the average body length retains utility as a holistic measure of the overall effects of metal ions on the population.

### Determination of population size and mortality in mixtures of Cd with different Zn concentrations

After 24 h the synchronous L1 nematodes were transferred to OP50 seeded NGM plates. 60 h later, approximately 10 adult nematodes were transferred to a 24-well plate filled with 1 mL K-medium (52 mM NaCl, 32 mM KCl, 5 μg/mL cholesterol, pH 5.1) [28] or test medium (K-medium containing the test metal concentration(s)), supplemented with *E*. *coli* OP50 (1.5 g/L). The well plates were continuously shaken (160 revolutions per minute (rpm), 20°C). Nematodes were exposed to 24 h LC2, LC5, LC20, LC40 and LC60 concentrations of Zn [23] with and without the presence of the LC20 concentration of Cd (Table 1). The population size (number of larvae and adults) was determined for each exposure over a period of 2 days. Nematodes were not transferred to a new well after 36 h, because of the short exposure period and the ability to correctly count the number of nematodes. Furthermore, after 0, 24, and 48 h of exposure, the number of live adult worms from the original population was counted in each well by the same procedure as described above.

### Statistical analysis

All statistical analyses were conducted with the program R, Version 3.1.2., with a 5% level of significance. Normality was checked visually by histograms and by the Shapiro–Wilk test. The Bartlett test was used to verify the homogeneity of variances.

#### Body length after LC5 and LC20 exposure

Linear mixed models were fitted to test the possible effects of exposure time, treatment and their interaction on the body length. For LC5 treatments, a linear model with time as a continuous variable was fitted. For LC20 treatments the change over time was not linear, thus time was entered as a categorical variable. Since body length was repeatedly measured over time, a random intercept term was added to account for the non-independence between observations within the same treatment.

For LC5, regression lines that model body length vs. time were fitted for each treatment separately. Multiple linear regression was used to compare the slopes of such lines for the single metals, the mixtures, and between the mixture and corresponding single metals. Significant differences in slopes were ascribed to differences in the time dependency of toxicity between treatments. A Dunnett test was carried out to compare the slopes of the treatments with the control.

In the nonlinear LC20 case, for each treatment separately, the difference in body length between the 3 time points was calculated with a one-way ANOVA with a Tukey correction for multiple comparison. Furthermore, for each day a one-way ANOVA followed by a Tukey post hoc test was fitted to determine the differences in body length between the single metals, the mixtures, and between the mixture and corresponding single metals. At both LC5 and LC20 levels, the results for the various metal treatments were compared with the control by a Dunnett post hoc test.

#### Population size after exposure to LC5, LC20 and ZnCd mixtures

Differences in population size, i.e. total number of live nematodes *N*, were analysed for LC5, LC20 and the various ZnCd mixtures separately. The presence of multiple generations and different trends in population size between treatments (exponential, linear, polynomial), made it difficult to describe the population growth rate by a single number and to compare the population growth rates between treatments. Therefore, for all combinations of treatments, three values that summarise effects on the *C*. *elegans* population size were compared, namely (i) the area under the population size vs. time curve, (ii) the maximum population size attained during the exposure, and (iii) the population size at the end point. For LC5 and LC20 treatments, the values were computed on the basis of the logarithm of the number of nematodes.

Areas under the population size vs. time curve were calculated using the trapezoid rule, as implemented in the trapz function using the pracma package in R [30]. This area is the cumulative sum of all individuals of the population multiplied by the time they were alive and is thus an indication of the population size during the whole experiment. Since treatments can have the same area under the population size vs. time curve, while showing a very different shaped curve, 2 more parameters (maximum and end population size) were measured to compare the patterns of population size between treatments.

The control group of the LC20 experiment was visually very different from the other treatments; since the focus was to compare differences between the various treatments, this control group was omitted from our analysis. One of the replicates of the LC20 concentration of Zn was also omitted, because it was an outlier according to diagnostic plots. Since the maximum population size of each LC5 and ZnCd mixture treatment was reached at the end of the experiment, only the end population size was analysed, except for the LC5 ZnCuCd mixture where the maximum was reached at day 8.

For each parameter value, a one-way ANOVA was fitted to test the effect of each treatment. To test if the magnitude of each value was different compared to the control, a posthoc analysis with a Dunnett correction was carried out. Subsequently, a one-way ANOVA was fitted for each parameter to test whether the outcome was the same across treatments. If there was a significant difference between treatments, a posthoc analysis with Tukey correction for multiple testing was performed. In this way, the differences between the single metals, between the mixtures and between the mixture and corresponding single metals were analysed.

#### Mortality of ZnCd mixtures

To determine whether the mixtures of LC20 concentrations of Cd with different Zn concentrations led to significant higher mortalities than the individual metals, a nonparametric ANOVA, Kruskal–Wallis test, followed by posthoc analysis with Bonferroni correction for multiple testing was performed.

Mixture effects were assessed using the conceptual model of Concentration Addition (CA) and Independent Action (IA) [31]. In contrast to population size, full dose-response profiles are available to predict mixture effects on mortality according to the more advanced formulas of CA and IA [23,32]. CA assumes similar modes of action, while IA assumes different modes of action of the single metals. The expression for the CA model is given by:

$$\sum_{i=1}^{n}\left(\frac{c_{i}}{\text{EC}50_{i}{\left(y/(100-y)\right)}^{1/\beta }}\right)=1$$ (1)

and that for the IA model is:

$$y=100\left[1-\prod_{i=1}^{n}\left(\frac{1}{1+{\left(c_{i}/\text{EC}50_{i}\right)}^{\beta }}\right)\right]$$ (2)

where *c*_*i*_ is the concentration of compound *i*, *n* is the number of compounds, *y* is the predicted effect of the mixture and *β* is the slope of the dose-response curve.

The observed mortalities were compared with the values expected on the basis of the CA model (Eq 1) and the IA model (Eq 2), using the non-parametric Wilcoxon test, to analyse which interactive effects occur in the mixtures. Additive effects were observed if predicted mixture effects did not deviate from measured effect. If observed mixture effects were significantly higher than those predicted from both models the interaction was considered as synergistic, whereas an antagonistic interaction was indicated by a significantly lower observed effect compared to that predicted by both models.

## Results and discussion

For clarity, we firstly discuss the effects of single metal exposures, followed by those of the various mixture exposures.

### Population effects of single metals: Metal treatment, concentration and time effects

#### Body length

**Fig 1:**
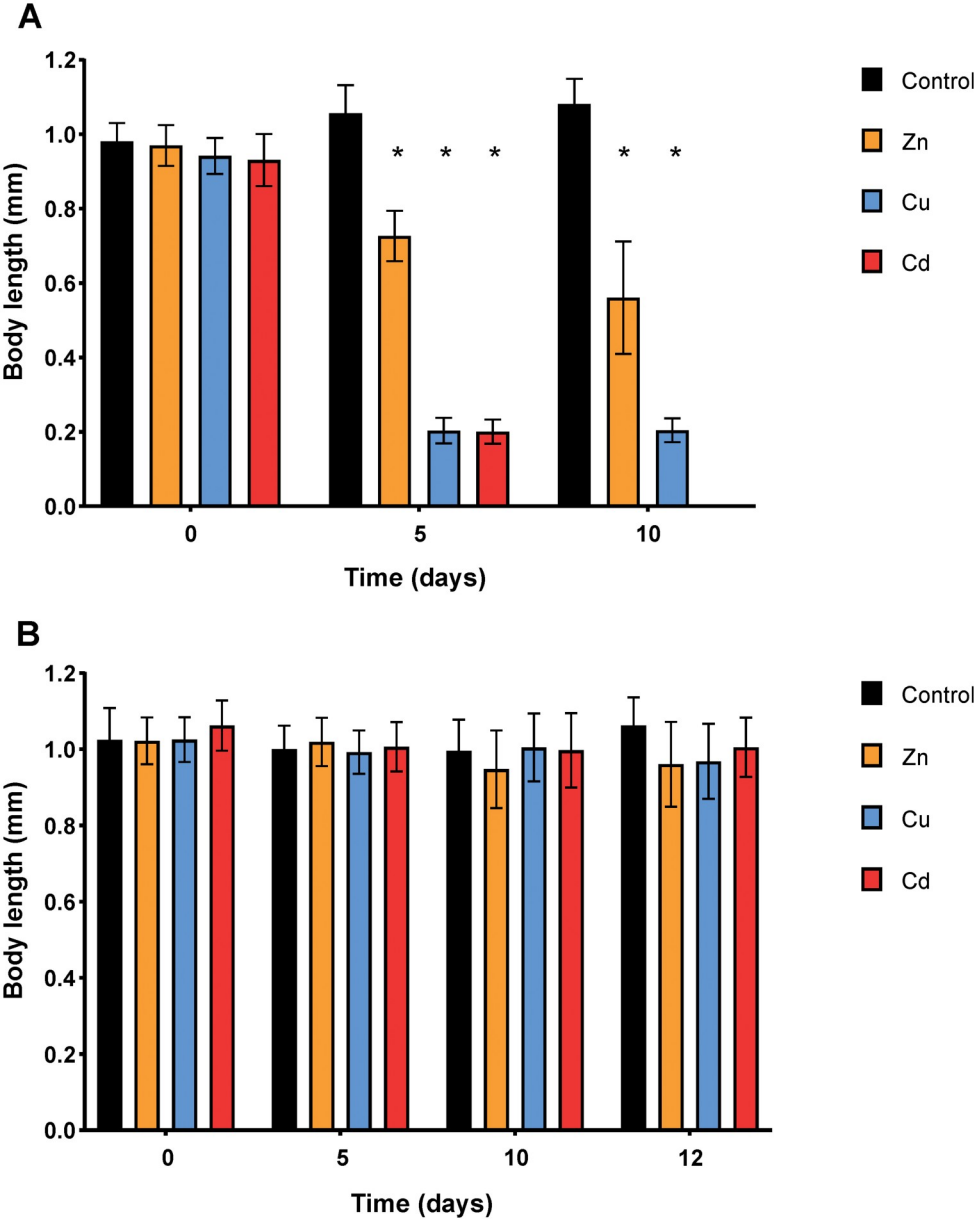
Body length (mm) of nematodes as a function of time (days) during exposures to single metal ions at LC20 (A) and LC5 (B) concentrations (see Table 1). Data are shown as mean ± standard deviation (20 individuals were measured for each of three independent replicates). Asterisks denote significant differences (*P*<0.05) compared to the corresponding control.

At the start of the experiments with LC20 concentrations of metal ions, all nematodes were adults of the same age (i.e. 24 h since onset of L4 stage) and body length as the control (Fig 1). As described in the Materials and methods, we did not follow the change in body length of particular individuals, but rather the average body length of the post-larval stage nematodes was determined and used as an indicator of effects at the population level.

The body length of nematodes exposed to LC20 concentrations of metal ions decreased over time, whilst that of the control organisms increased (*P*<0.001) (Fig 1A; S2 Table). After 5 days of exposure, the Cu and Cd exposed nematodes were 80.9% smaller than the control, while for Zn exposures they were 31.2% smaller (*P*<0.001) (S3 Table). The maximum measured body length of Cu and Cd exposed nematodes corresponded to the length of L1 larvae, while Zn exposure resulted in a similar length as L4 larvae. At the end of the experiment (day 10), the difference in body length between the control and Cu exposed nematodes was similar to that of day 5, while the difference compared to Zn (+17.3%) was larger (*P*<0.001) (S4 Table). Cu exposed nematodes had a similar maximum size as at day 5, while the maximum measured length of Zn exposed *C*. *elegans* was reduced to the size of L3 larvae at day 10. Since the nematodes exposed to Cd were dead at the end of the experiment, their body length was not measured. It is possible that exposure to single metal ions at LC20 concentrations affected the development of *C*. *elegans*, resulting in smaller body lengths. In contrast, the body length of LC5 exposed nematodes was not affected by any of the single metal treatments (Fig 1B), which suggests that they reached adulthood.

These observations are in agreement with trends reported in earlier studies. For example, the EC50 for body length after an exposure of 24 h was 0.05 mM (3.2 mg/L) for Cu, 0.23 mM (15.1 mg/L) for Zn, and 0.05 mM (5.7 mg/L) for Cd [33]. Others have observed a concentration dependent effect of Cd on developmental stages, e.g. exposure to 20 μM (2.3 mg/L) Cd decreased the number of adults and increased the proportion of nematodes in the L4 stage with a very small number of nematodes in the L3 stage [17]. Mixed populations of L4 and L3 larval stages were present after exposure to 30 μM (3.4 mg/L) Cd, while no nematodes developed further than L1-L2 larval stages when exposed to 100 μM (11.2 mg/L) Cd. In the case of Cu (on NGM plates), at the optimal Cu concentration of 2 μM (0.13 mg/L), *C*. *elegans* developed at day 3 into gravid adults, whereas at higher Cu concentrations nematodes maximally reached L3 larval or young adult stage [34]. Similarly, in our study nematodes exposed to Cu at LC5 (ca. 4 μM; 0.23 mg/L) developed into adults, while at LC20 (ca. 20 μM; 1.3 mg/L) they only reached the length of L1 larvae. In the case of Zn (in *C*. *elegans* maintenance medium (CeMM)) nematodes were not able to mature or reproduce in the absence of Zn; exposure to 1–10 μM (0.07–0.65 mg/L) caused impaired growth, and optimal growth was in the range of 30 μM to 1 mM (1.96–65.39 mg/L) [9]. In the present study (in liquid medium) body length was not affected by 22 μM (1.4 mg/L) of Zn, but was reduced upon exposure to 145 μM (9.5 mg/L) Zn. Differences between studies in terms of the absolute concentrations at which effects were observed can be ascribed to the different test media and differences in protocols, amount of bacteria, and type of bacteria etc. These effects of single metal treatments on development of LC20 exposed nematodes are reflected in the observations of population size discussed below.

#### Population size

**Fig 2:**
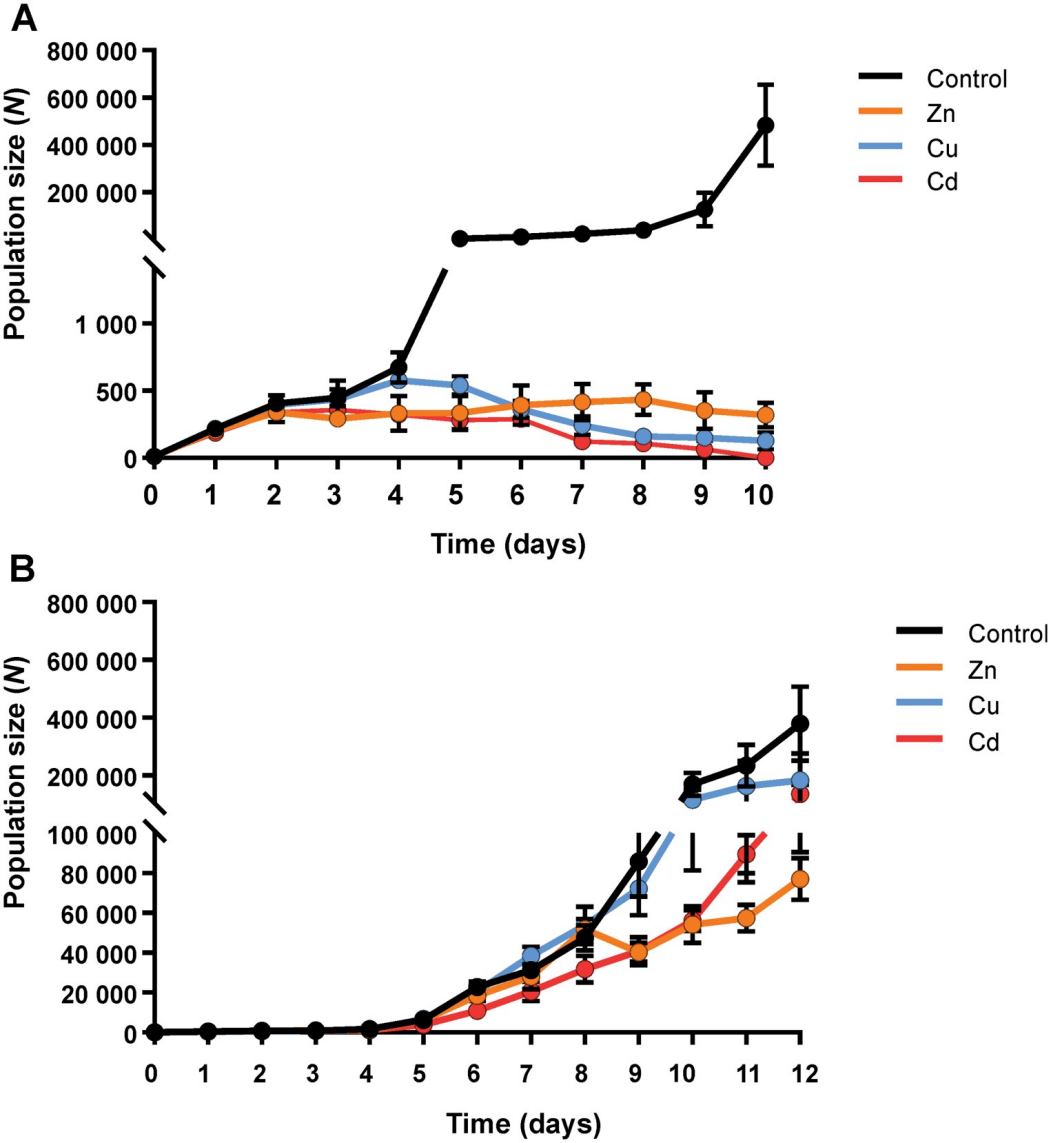
Population size (number, *N*) of nematodes as a function of time (days) during exposures to single metal ions at LC20 (A), and LC5 (B) concentrations. Data are shown as mean ± standard deviation (number of independent replicates, *n* = 3). The starting population in all treatments is 10 adult nematodes.

For control nematodes, the start of new generations was observed clearly with a population increase every 3–4 days. Nematodes exposed to LC20 concentrations of single metals did not show this pattern (Fig 2A). Instead, it seems that only one new generation was formed, whereafter the population size started to decrease. Zn had a similar maximum population size as Cu, although this was already reached at day 4 for Cu, while the population size of Zn slightly increased until day 8. Thereafter, compared to the respective maximum population sizes, the end population size decreased by 25.9% for Zn and by 77.8% for Cu exposure. At day 10, the population size of the Zn and Cu exposed nematodes was comparable. Following attainment of the maximum population size (day 3), the population of Cd exposed nematodes decreased dramatically until all were dead at day 10 (Fig 2A). Thus, as the exposure time increased, the toxicity of Cd was increasingly manifested. Furthermore, Cd had a smaller area under the population size vs. time curve, smaller maximum and smaller end population size compared to Zn and Cu (S5, S6 and S7 Tables). Further information on the population size and area under the population size vs. time curve for the LC20 treatments is given in the Supplemental data, S5, S6 and S7 Tables. The crucial role of the exposure time on the relative magnitudes of the observed effects points to differences in the dynamics of uptake and biotic handling processes amongst the three metals [35].

When exposed to the lower LC5 concentration, Cd caused a lower area under the population size vs. time curve (58.9%, *P*<0.05) (Fig 2B; S8 Table), while Zn caused a lower maximum population size (78.3%, *P*<0.01) compared to the control group (S9 Table). Interestingly, the population size in the Cu exposure did not differ from the control, suggesting that the chosen Cu concentration might approximate the essential Cu concentration [34]. Nevertheless, upon extending the exposure time beyond day 10, the population size in the Cu exposure levelled off from day 11 onwards, whereas the control population further increased (Fig 2B). This finding shows that studies with longer exposure times are necessary to reveal the potential toxicity of low Cu concentrations in the longer term. Population sizes in single metal exposures to Zn and Cd were similar until day 10, after which the population of Cd increased to a greater extent and approached that of Cu at day 12. In contrast to the LC20 exposures, the maximum population size of all single metal treatments was reached at day 12.

Several studies have reported the effects of single metal exposures on *C*. *elegans* at the population level which are in broad agreement with our findings [36,37]. For nematodes exposed to LC20 concentrations of metal ions, a decrease in population size was expected because the nematodes did not reach adulthood, which disrupted egg laying, causing the number of new larvae to decline. Furthermore, larval nematodes are more sensitive to metal toxicity than adults [21], which also contributes to the observed population size decrease. Exposure time was also found to be an important factor in determining the absolute and relative magnitude of toxic effects. Short-term 24 h or 48 h exposures typically show Cu to be more toxic than Zn or Cd to *C*. *elegans* [20–24]. However, in the present study, the relative toxicity order of Cu and Cd was reversed as the exposure time increased. This effect has been reported by others on e.g. mortality [24].

Although similar declining trends were observed for body length and population size of nematodes exposed to LC20 concentrations of metal ions, no effects on body length were observed after exposure to LC5 concentrations, while population size was slightly affected. It seems that the reproductive characteristics (start of egg laying, number of eggs, time of hatching, hatching success, etc.) of the nematodes exposed to LC5 concentrations of metal ions were affected, which is reflected in differences in population size. These observations indicate that population size is a more holistic and sensitive endpoint than body length alone.

The hypothesis that an affected development and reproduction may play an important role in the declining population sizes of nematodes exposed to LC5 and LC20 concentrations of metal ions has been confirmed in earlier studies. According to numerous publications, the fecundity and growth of *C*. *elegans* is reduced by Cd [13,18,38,39]. Decreased fecundity is ascribed to a combination of slow growth and shorter lifespan, preventing development into adulthood, combined with a decrease in number of eggs produced of which fewer were fertilised [40]. These results are in agreement with findings for isopods, *Daphnia magna* and mites [36,38,40–42].

Although Cu is essential, in excess or deficiency it has detrimental effects on brood size and life span and causes an increase in generation time and impairment of development (manifested as reduced body size and growth) [4,16,23,34,43,44]. Since Cu affects reproduction and life-span, it consequently influences population growth. The reproduction EC50, i.e. the concentration of Cu required to reduce reproduction by 50% relative to control, was found to be 0.032 mM ± 0.003 mM (2.04 ± 0.19 mg/L) for *C*. *elegans* and 0.035 mM ± 0.015 mM (2.21 ± 0.93 mg/L) for *P*. *pacificus* [8]. Furthermore, the population growth at day 3 was maximal at low Cu concentrations (≤ 10 μM; ≤ 0.6 mg/L), but impaired at 150 μM (9.5 mg/L) Cu, causing smaller brood size and delayed development [34]. These results are consistent with the present study: the population exposed to the LC20 concentration of Cu (20 μM), reached its maximum size at day 4 and thereafter decreased, which suggests that the reproductive traits were affected.

In the case of Zn, deficient or excess concentrations are known to have harmful effects on (population) growth, survival, development and reproduction of many animals, including *C*. *elegans*. [29,45–47]. Besides population size, lifespan decrease has also been reported to be concentration dependent: a Zn concentration of 2.5 μM (0.16 mg/L) caused a lifespan shortening of 3 days, while exposure to 75 μM (4.90 mg/L) or 200 μM (13.08 mg/L) reduced lifespan by 4 days [29].

Overall, single metal treatments of Zn, Cu and Cd are all able to influence life span and several characteristics, which could explain the observed effects on body length and population size. In addition, hormesis (an adaptive response to low stress levels) might explain the relatively large population size after LC5 exposures in the present study [48].

### Population effects of mixtures: Metal treatment, concentration and time effects

#### Body length

**Fig 3:**
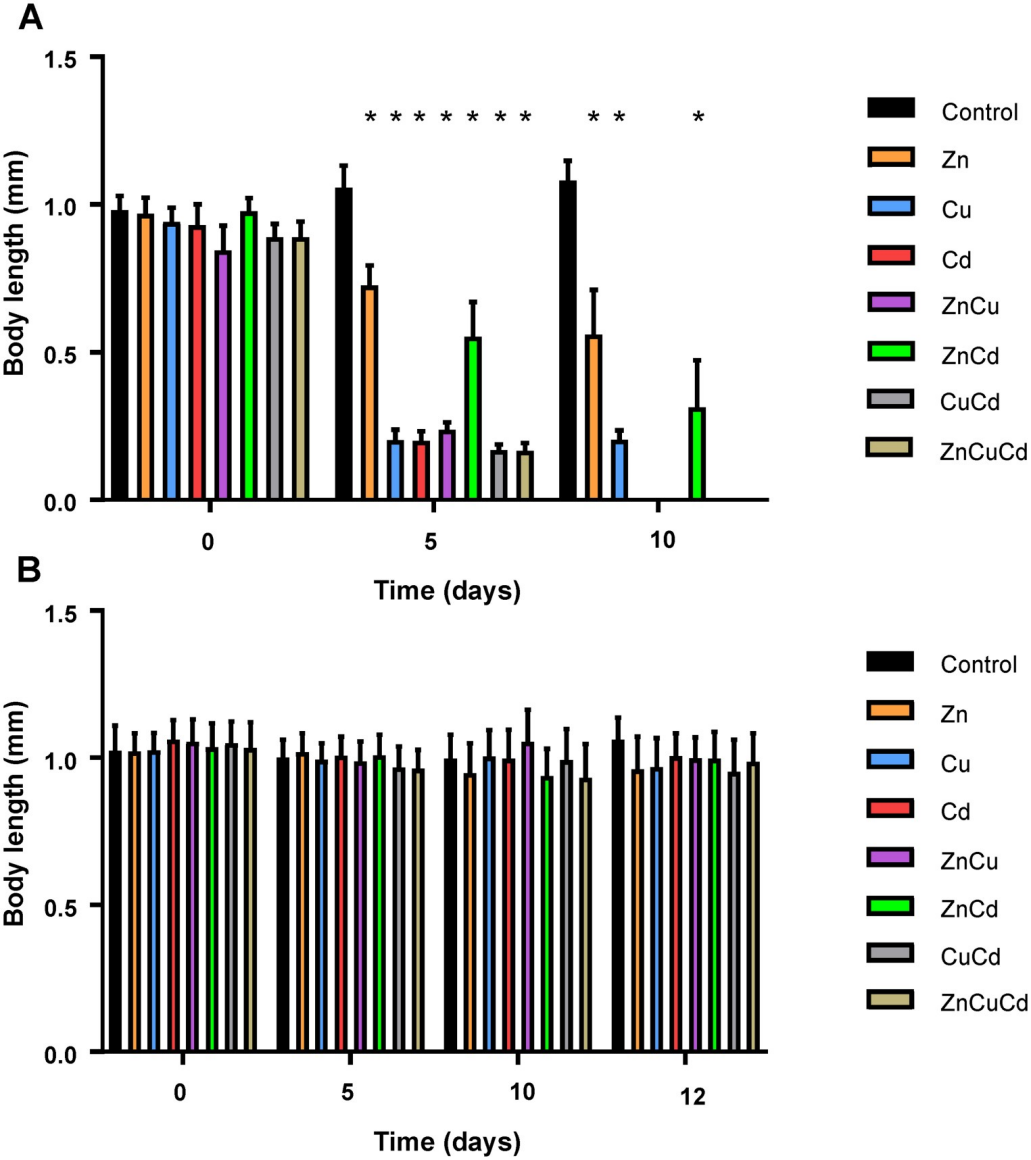
Body length (mm) of nematodes as a function of time (days) during exposures to mixtures of metal ions at their respective LC20 (A) and LC5 (B) concentrations (see Table 1). Data are shown as mean ± standard deviation (20 individuals were measured for each of the three independent replicates). Asterisks denote significant differences (*P*<0.05) compared to the corresponding control. The single metal data are included for comparison and are the same as those shown in Fig 1.

The body length of nematodes exposed to mixtures of LC20 concentrations of metal ions decreased over time (Fig 3A, S2 Table). After 5 days of exposure, the ZnCu, CuCd and ZnCuCd exposed nematodes were 77.5–84.2% smaller than the control, while for ZnCd exposure they were 47.6% smaller (*P*<0.001) (S3 Table). After exposure to ZnCd, the body length was much larger compared to the other mixtures and 175.6% larger than of those exposed to Cd alone, while body length of nematodes exposed to Zn was 31.3% larger than of those exposed to ZnCd (*P*<0.001). This suggests that Zn reduced the toxic effect of Cd on the body length of *C*. *elegans*. In contrast, the body length of nematodes exposed to ZnCu was similar to that for exposures to Cu alone and much smaller than for Zn alone (*P*<0.001) (S3 Table). Apparently Zn was not able to mitigate Cu toxicity, rather it seemed to increase the toxicity. The body length of nematodes exposed to Cu and Cd alone was on average 19.6% larger compared to CuCd (*P*<0.01). The tertiary mixture led to a smaller body length compared to any of the single metals. At the end of the experiment (day 10), the difference in body length between the control and ZnCd (+23.4%) was larger (*P*<0.001) than at day 5 (S4 Table). Nematodes exposed to ZnCu, CuCd and ZnCuCd were dead, therefore their body length was not measured. The body length of Zn was 78.3% larger than ZnCd at day 10 (*P*<0.01) (S4 Table). As discussed in the preceding sections for the single metals, exposure to mixtures of metal ions at LC20 concentrations might also affect the development of *C*. *elegans*, causing smaller body lengths. This was reflected at day 5 in the correspondence between the body length of ZnCu, CuCd and ZnCuCd exposed nematodes and the length of L1 larvae, while the body length of nematodes exposed to ZnCd was similar to that of L3 larvae. At day 10, the body length of nematodes exposed to ZnCd was similar to the length at the first molt (L1 to L2 larvae) or of dauer larvae. These results indicate that nematodes exposed to mixtures of metal ions at LC20 concentrations were not able to reach adulthood, leading to a decline in population size.

In contrast, the body length of nematodes exposed to mixtures of metal ions at LC5 concentrations was similar to the control and the single metal exposures (Fig 3B).

#### Population size

**Fig 4:**
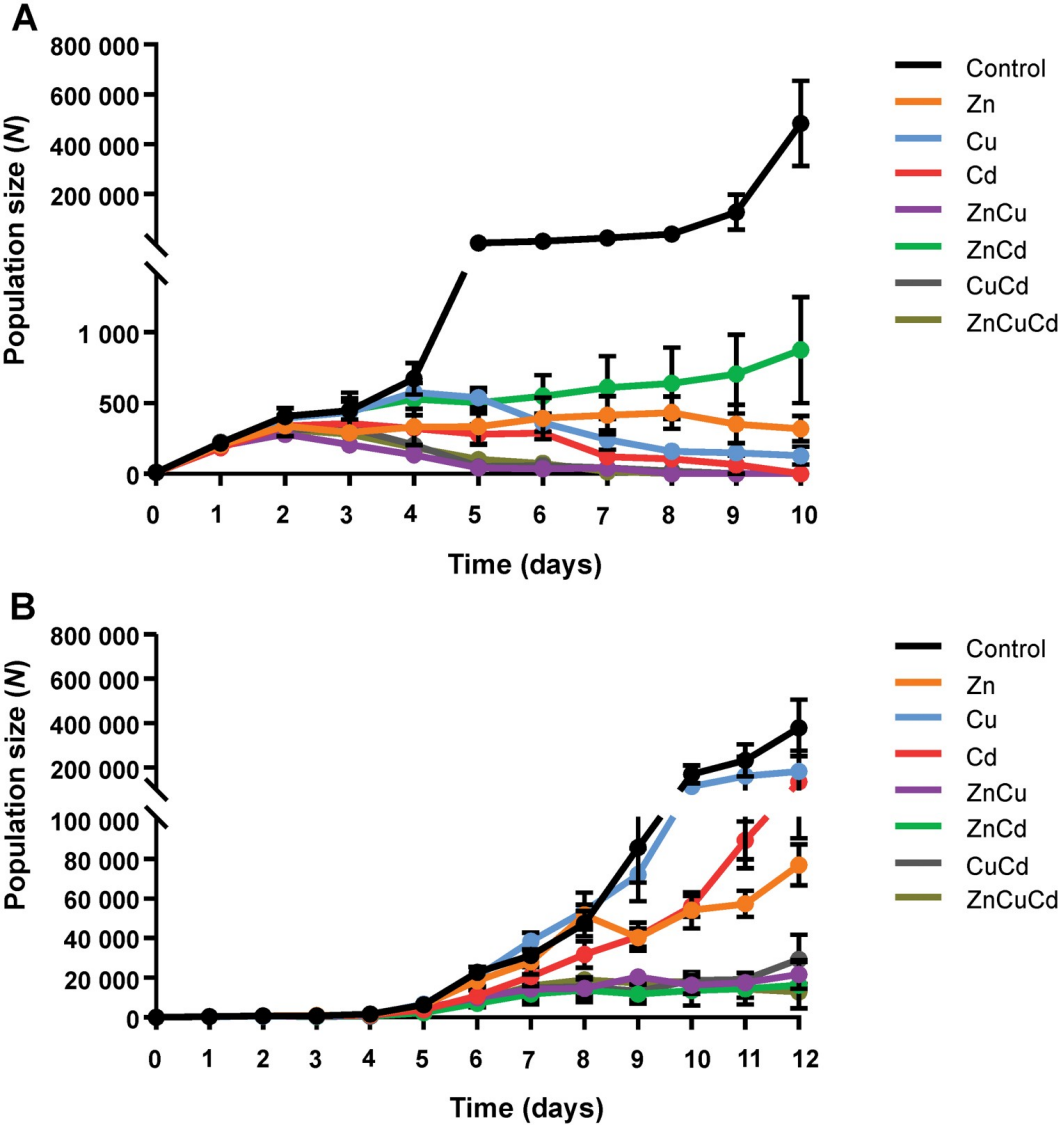
Population size (number, *N*) of nematodes as a function of time (days) during exposures to mixtures of metal ions at their respective LC20 (A), and LC5 (B) concentrations. Data are shown as mean ± standard deviation (number of independent replicates, *n* = 3). The starting population in all treatments is 10 adult nematodes. The single metal data are included for comparison and are the same as those shown in Fig 2.

The maximum population size of nematodes exposed to ZnCu, CuCd and ZnCuCd mixtures at their respective LC20 concentrations was reached within 2–3 days and no nematodes survived until the end of the experiment at day 8–9 (Fig 4A). These mixtures led to a lower area under the population size vs. time curve, a lower maximum population size and a decreased population size at day 10 of exposure as compared to at least one of the corresponding single metals (S5, S6 and S7 Tables). Interestingly, the nematodes exposed to ZnCd had a larger area under the population size vs. time curve and larger population size at day 10 compared to Cd alone, and a larger area under the curve, larger maximum and greater end population size than the other mixtures (S5, S6 and S7 Tables). The maximum population size of the ZnCd exposed nematodes was reached at day 10. Zn therefore seemed to have a mitigating effect on the toxicity of Cd.

In contrast to the LC20 treatments, the maximum population size of all combinations of metal ions at their respective LC5 concentrations was reached at day 12, except for ZnCuCd where the maximum was reached at day 8. Nematodes exposed to the LC5 combinations ZnCu, ZnCd, CuCd and ZnCuCd (86.9%, *P*<0.001) had a lower area under the population size vs. time curve and lower end population size (94.8%, *P*<0.001) than the control group (Fig 4B; S8 and S9 Tables). Furthermore, all LC5 mixtures had a lower area under the population size vs. time curve and smaller end population as compared to the corresponding single metals (Fig 4B, S8 and S9 Tables). In contrast to the LC20 exposure, the population size in the LC5 ZnCd mixture had a similar pattern to that of the other mixtures. It seems that the LC5 concentration of Zn was too low to mitigate toxicity effects at the population level. For all mixtures of metal ions at their respective LC5 concentrations, no deleterious declines in the number of living nematodes were noticed, rather the population size of mixtures even slightly increased over the course of the exposures (Fig 4B).

The interactive effect of metal ions on population size in mixtures of ZnCu, CuCd and ZnCuCd at both LC20 and LC5 concentrations seemed to be either additive or synergistic. The differences in toxicity indicate the importance of simultaneous testing of mixtures and the corresponding single metals. Furthermore, it is important to take exposure time into account, since the nature of the interactive effects may evolve with time [49]. In our study, mixtures of metal ions at their respective LC20 concentrations did not differ from single metals in terms of when the maximum population size was reached (Fig 4A). However, thereafter, the population size of mixtures decreased much faster compared to the corresponding single metals. Even for LC5 levels, differences in population size between mixtures and single metals were more obvious from day 6 onwards (Fig 4B). Furthermore, it is possible that additivity is only noticed under certain conditions. In our study the combination of Cu and Cd had a larger additive effect when both metal ions were present at LC5 concentrations than at LC20 ones, while the combined additive effect of Cu and Zn each at LC5 concentrations was similarly additive or slightly less additive than at LC20 (Fig 4). However, in the study of Jonker et al. [31] synergistic interaction effects of Zn and Cu on the population growth, after 1 week of exposure, were only observed at high dose levels (i.e. higher than LC50). In contrast, the interaction between Zn and Cd in the present study appeared to be antagonistic at LC20 concentrations of each metal ion; and additive at LC5 concentrations.

The toxic effects were again more evident for population size than for body length. For example, after exposure to the ZnCd mixture of LC20 values, body length was on average 196.6% larger as compared to the other mixtures (Fig 3A), while population size was on average 768.0% higher (Fig 4A). The differences between mixtures and the corresponding single metal exposures were also more apparent for population size than for body length. At day 5, the population sizes of Cu and Cd exposed nematodes were on average 707.9% larger than of those exposed to the CuCd mixture, while their body length was on average only 19.6% larger (*P*<0.01).

Although metal mixture toxicity is typically evaluated in terms of the “concentration addition” (CA) model or the “independent action” (IA) model, these simplistic additive approaches have limitations [32,50,51]. For example, mixture toxicity effects on the temporal evolution of the body length and population size of *C*. *elegans* are difficult to interpret with this simple framework. As noted above, the body length does not refer to a particular individual but rather represents the average body length of the post-larval stage nematodes of the population, which are not necessarily the same age or from the same life stage. These conditions, as well as the absence of full dose-response profiles, preclude use of the available expressions for the CA and IA models for analysing mixture effects. As a next step, additional experiments with different exposure concentrations are needed to fit dose-response curves as a function of exposure time, and to use this information to model and predict mixture effects at these organisational levels. Such studies would allow an evaluation of the ability of the CA and IA models to predict the temporal evolution of mixture effects. However, these models should be applied with some caution. Our results showed a different population size pattern for LC20_24h_ and LC5_24h_ exposures, which changed over time. Given these limitations, the relative toxicity effects exerted by mixtures on body length and population size were described and compared with those arising from the corresponding single metals. In most cases the interactive effects are evident from the data shown in the figures, e.g. antagonistic effect of Zn and Cd in LC20 mixtures (Figs 3 and 4).

Mixtures of metals at LC5 concentrations had no effect on body length, while ZnCu, CuCd and ZnCuCd mixtures at their respective LC20 concentrations seemed to cause either additive or synergistic effects. These mixtures of both LC5 and LC20 concentrations also caused either additive or synergistic effects on population size. Others have reported that Zn and Cu have additive or antagonistic effects at the individual level (mortality, behaviour) and at the community level (number of nematodes in soil) [23,52], and that there are highly synergistic interactions between Cu and Cd, and between Cu and Zn at the individual level (mortality) [21]. Such interactive effects were also observed at the molecular level in a transgenic strain of *C*. *elegans*, carrying a stress-inducible β-galactosidase reporter, where the combination of Cu and Cd in soil led to a larger response than Cd alone, whereas the ZnCd mixture caused a lower β-galactosidase activity than Cd alone [16]. This antagonistic effect was also observed in the present study for the LC20 combination on both body length and population size. Zn seemed thus to have a mitigating effect on Cd due to e.g. competition for Ca channels. Furthermore, this effect was also observed in our previous work at the individual level (mortality and locomotion) [23] and in other studies at the molecular (β-galactosidase activity) and individual level (mortality, body burden, body length) [13,21].

### Mitigating effect of Zn on Cd toxicity at the individual and population level

**Fig 5:**
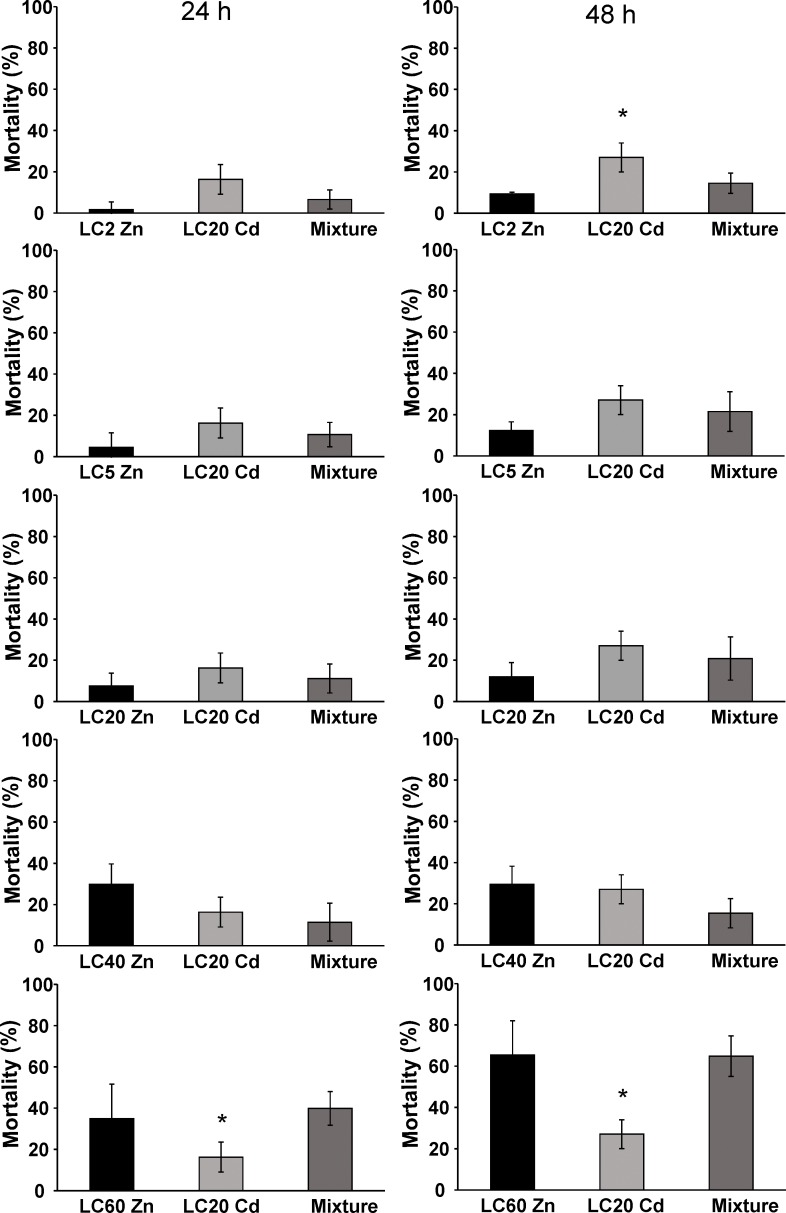
Mortality (%) of nematodes exposed to 24 h lethal concentrations LC2, LC5, LC20, LC40, LC60 of Zn, LC20 of Cd, and respective mixtures thereof. Nematodes were exposed for 24 h (left) and 48 h (right). Data are shown as mean ± standard deviation (number of independent replicates, *n* = 6). Asterisks (*) denote significant differences (*P*<0.05) compared to the mixture. See Table 1 for LC_24 h_ values.

To better understand the mitigating effect of Zn, the population size and mortality of a range of LC concentrations of Zn in combination with a LC20 concentration of Cd was measured. All combinations at 24 h and 48 h, had a lower mortality than that predicted on the basis of the CA and IA models (*P*<0.05), i.e. antagonistic effects are operative (Fig 5, S10 and S11 Tables). Furthermore, compared to the exposure to Cd-only at the LC20 concentration, the mixture of LC60 Zn + LC20 Cd at both time points, caused a significantly higher mortality, while the mixture LC2 Zn + LC20 Cd at 48 h induced a lower mortality (S10 and S11 Tables).

**Fig 6:**
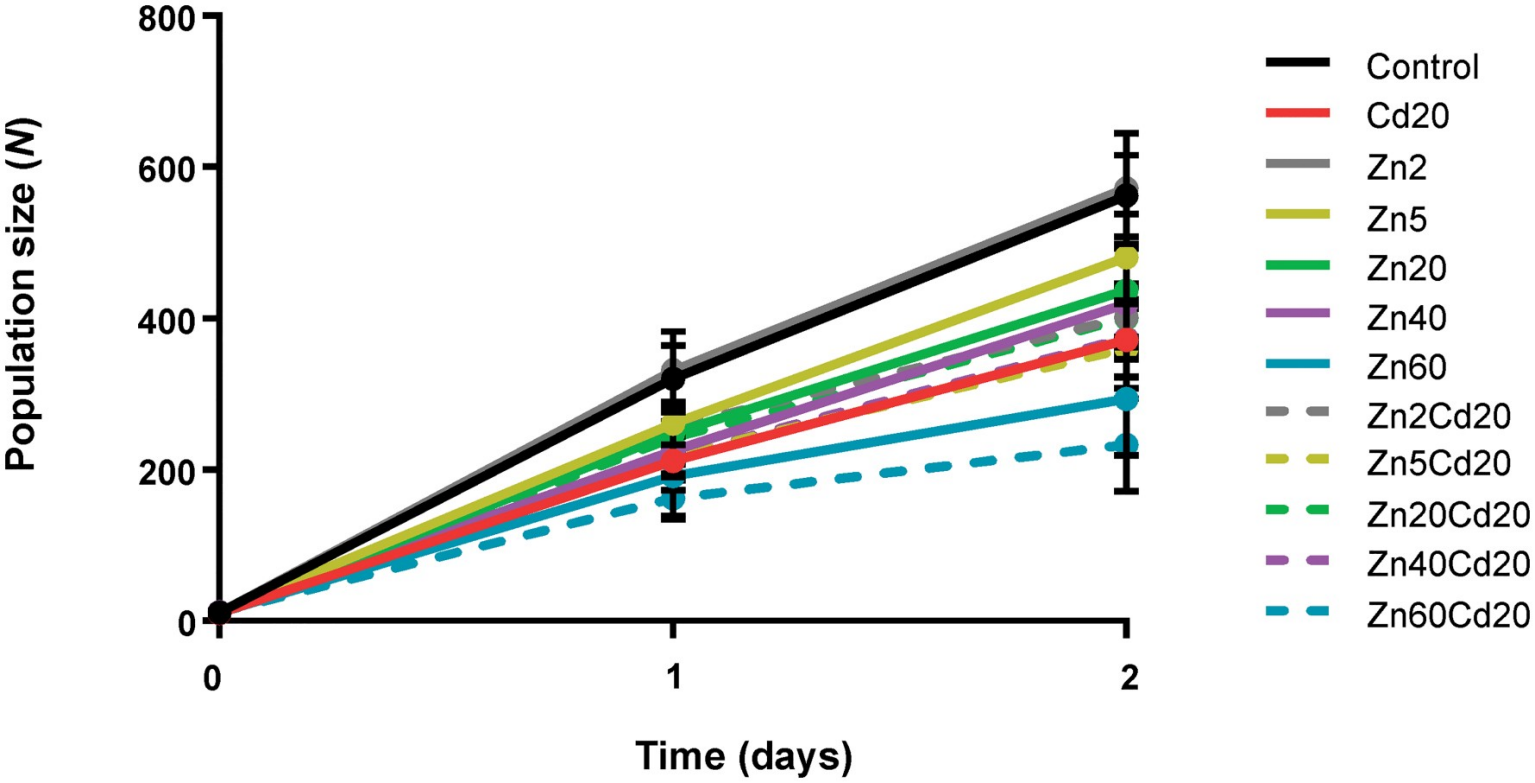
Population size (number, *N*) of nematodes as a function of time (days) during exposure to Zn at a range of LC levels, both singly and in combination with an LC20 concentration of Cd. Data are shown as mean ± standard deviation (number of independent replicates, *n* = 3). The starting population in all treatments is 10 adult nematodes.

Although all ZnCd combinations had an antagonistic effect on the mortality, this effect seemed to be concentration dependent and the strongest antagonistic effect was observed for the mixture of LC40 Zn + LC20 Cd. This concentration dependency of the mitigating effect of Zn was more pronounced for the population size. Population size for all treatments reached its maximum at the end of the experiment, day 2, even for the mixture LC60 Zn + LC20 Cd for which a high mortality was noted, but a concentration dependent relation was observed (Fig 6). When an LC20 concentration of Cd was combined with an LC2 concentration of Zn (*P*<0.001) or an LC5 concentration of Zn (*P*<0.01), the population size decreased, resulting in smaller areas under the population size vs. time curve and smaller end population sizes compared to the corresponding Zn-only exposures (S12 and S13 Tables). Furthermore, the combination of an LC60 concentration of Zn with an LC20 concentration of Cd resulted in a significantly smaller area under the population size vs. time graphs (*P*<0.05) and end population size (*P*<0.01) compared to Cd-only at its LC20 concentration. In contrast, the mixtures LC20 Zn + LC20 Cd and LC40 Zn+ LC20 Cd had a population size that was similar to the corresponding single metal exposures, suggesting that Zn concentrations in this range were best able to mitigate the toxicity of LC20 concentrations of Cd. Apparently, LC5 and LC2 concentrations of Zn were too low to protect the nematodes against the harmful effect of Cd, while LC60 of Zn alone already caused a population decline of 46.7%. The concentration dependence of Zn toxicity is apparent from data reported by Dietrich et al. [12]: in CeMM liquid medium, the population growth rate was maximal at Zn concentrations ranging from 30–500 μM, dose-dependent decreases were noticed for concentrations higher than 500 μM and the population growth rate was severely impaired at 2.5 mM Zn. In our study, the population size also decreased dose-dependently, especially LC60 Zn (1.3 mM) caused a significant reduction (Fig 6). Population size was maximal at concentrations below 30 μM. This suggests that high Zn concentrations disturb Zn homeostasis and that excessive Zn can replace other physiologically important metal ions such as Cu and Mn, or bind to ectopic protein sites [12]. The concentration-dependent protective role of Zn on Cd toxicity has been reported in studies on a wide range of organisms including *Daphnia magna* [53–55], freshwater bivalves [56], cladocera [57] and rainbow trout [58].

**Fig 7:**
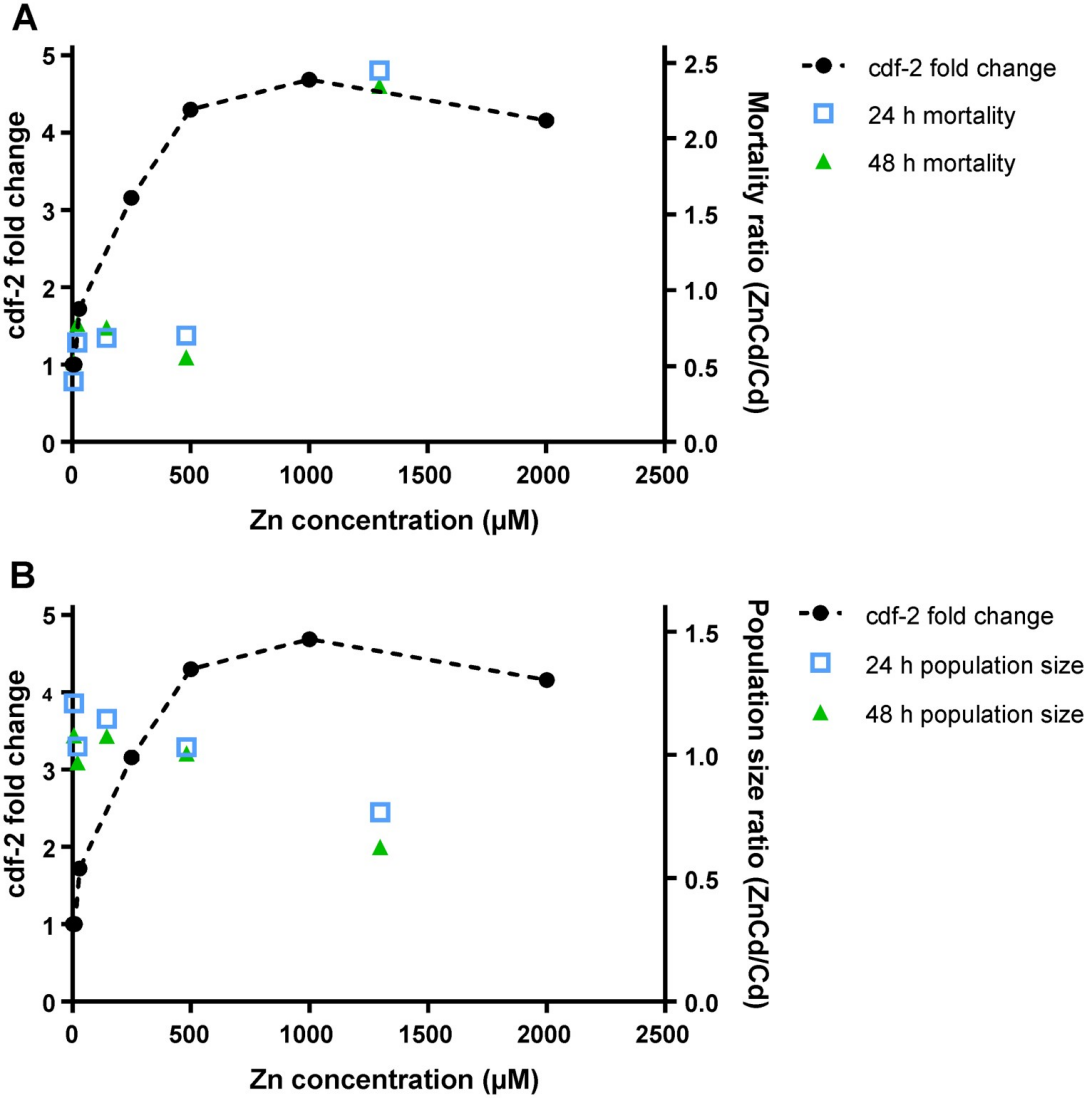
**(A) Effect of Zn on the mortality induced by a LC**_**24 h**_**20 concentration of Cd after 24 h and 48 h (right-hand axis) as compared to the fold-change in level of *cdf-2* expression in Zn-only exposures (left hand axis; data from Davis et al., 2009 [9]).** The mortality data are shown as the ratio of mortality in the ZnCd mixture to that in the corresponding Cd only exposure, i.e. a value greater than unity reflects greater toxicity in the mixture exposure. The fold-change in *cdf-2* expression is relative to that observed in Zn at 2 μM. **(B) Effect of Zn on the population size of nematodes exposed to an LC20**_**24 h**_ **concentration of Cd after 24 h and 48 h (right-hand axis) as compared to the fold-change in level of *cdf-2* expression in Zn-only exposures (left hand axis).** The population data are shown as the ratio of the population size (number, *N*) in the ZnCd mixture to that in the corresponding Cd only exposure, i.e. a value less than unity reflects greater toxicity in the mixture exposure.

Zn and Cd are both Group 12 metals that can bind to identical macromolecular structures via coordination with oxygen, nitrogen and sulphur functionalities, albeit with different affinity [59,60]. In general, Cd can replace Zn in various proteins causing dysfunction and can compete with Zn as a cofactor. Cellular detoxification systems (including glutathione, metallothioneins, heat shock proteins, pumps and transporters) regulate intracellular metal levels by detoxifying and excreting metals [10]. In *C*. *elegans* transmembrane Zn transporters belong to the CDF or ZIP family. ZIP proteins increase the concentration of cytoplasmic Zn by importing, while CDF proteins function to lower cytoplasmic Zn concentration by exporting across the plasma membrane [11,12]. Amongst members of the CDF family, *cdf-1* seems to promote resistance to high Zn levels by promotion of Zn excretion and/or limiting Zn uptake, while *cdf-2* functions to promote Zn accumulation. High levels of Zn induce transcript levels of *cdf-2*, *ttm-1b*, *mtl-1* and *mtl-2*, the promoters of which contain a similar sequence, called HZA [11,12]. It is suggested that in *C*. *elegans* HZA serves as an enhancer to mediate transcriptional activation in response to high Zn or Cd levels [11,12]. In contrast, Cu does not affect gene expression, indicating that HZA does not mediate a response to all metals [11,12]. In the study of Davis et al. [9] a dose-response increase in *cdf-2* expression was noticed as a function of increasing Zn concentration, while *cdf-1* was most abundant at low Zn concentrations. We compared the fold-change in *cdf-2* expression reported by Davis et al. [9] with our observed changes in mortality and population size. Remarkably, Fig 7 shows a consistency between the literature gene transcription data [9] and our mortality results. Specifically, beyond a certain concentration of around 1 mM of Zn (i.e. around the LC60 concentration of Zn in our study), no further increase in *cdf-2* expression was reported [9] and this corresponds to the condition under which we observed a decrease in the extent of antagonistic effects in the ZnCd mixtures.

It is possible that the mixture of LC60 Zn + LC20 Cd reached or even exceeded the capacity of the system to cope. That is, above a certain Zn concentration there is no further increase in the ability to excrete, chelate or store Zn or Cd, and thus toxicity of both metals is manifested. At the other extreme, i.e. limiting Zn conditions, it has been reported that Cd can displace Zn and trigger a transcriptional response that results in reduced Zn availability [11,12]. This process may explain aspects of the mode-of-action of Cd toxicity and its time dependence.

The overall toxicity observed at the individual and population levels is a consequence of the dynamic features of all the involved components (uptake, internal handling and elimination). The translation of exposure to accumulation and toxicity requires knowledge of the kinetics of all the contributing events. The HZA element appears to provide important insights into mechanistic links between the effects of Zn and Cd at the gene transcription level and those at the individual and population level.

## Conclusions

Our study confirmed that metal toxicity to *C*. *elegans* is concentration and time dependent, resulting in different trends for population size and body length. In turn, different trends were observed under single vs. mixed-metal exposure conditions. The nature and extent of the effects observed under mixture scenarios were also dependent on the metal combinations employed. Nevertheless, with the exception of ZnCd combinations, the toxicity of mixtures was always greater than that of the corresponding LC concentrations of single metals, and thus either additive or synergistic. The mitigating effect of Zn on Cd toxicity was found to be concentration dependent: e.g. an LC20 concentration of Zn reduced the toxicity of an LC20 concentration of Cd, whilst an LC5 concentration of Zn had no effect.

A significant outcome of our study is the importance of studying effects on different endpoints and on different organisational levels, and the time dependence thereof. Toxic effects at one level, e.g. the molecular or individual, can help to better understand the observations at another organisational level, e.g. the population. Furthermore, it is possible that at a certain time point no effects are seen at one level, but are evident at another. These findings underscore the need to monitor effects at multiple organisational levels. This study also highlighted the importance of long-term studies. For example, differences between LC20 treatments can already be observed for mortality and behaviour after 24–48 h [23], while for population size differences became more evident after 3–4 days of exposure. This can be explained by the fact that population size integrates individual life histories and transgenerational effects, which take time to be reflected in the whole population. For example, if the development of nematodes of the new generation is affected, the effects will only be clearly visible 3–4 days later via the absence of a large increase in population size due to e.g. inability to lay eggs. Overall, metal effects were more evident on population size rather than body length or mortality, suggesting that population size is a sensitive endpoint for effects on long timescales.

Overall, it is evident that data on metal (mixture) effects at different levels (molecular, survival, reproduction, population, etc.) and exposure timescales is needed to elucidate the mechanistic basis of toxic effects. Unfortunately, environmental quality standards are still based on single metal toxicity at short exposure durations and do not take into account the chronic, multi-stressor exposure scenarios that are typical for environmental systems. The consistency we observe herein between gene transcription responses and mixed metal toxicity at the individual and population level may lead the way to development of a more comprehensive basis for environmental risk assessment.

## Supporting information

S1 Table Dilution protocols for counting nematodes in LC5 and LC20 treatments. (XLSX)

S2 Table Body length changes over time during LC20 exposures. Percentages represent the increase in body length over time for control and decrease over time for metal treatments. Asterisks denote significant differences (*P<0.05; ** *P*<0.01; *** *P*<0.001) (ns = not significant; NA = not applicable).
(XLSX)

S3 Table Between treatment comparison of the body length after 5 days of exposure to LC20 concentrations. Asterisks denote significant differences (*P<0.05; ** *P*<0.01; *** *P*<0.001) (ns = not significant; NA = not applicable).
(XLSX)

S4 Table Between treatment comparison of the body length after 10 days of exposure to LC20 concentrations. Asterisks denote significant differences (*P<0.05; ** *P*<0.01; *** *P*<0.001) (ns = not significant; NA = not applicable).
(XLSX)

S5 Table Between treatment comparison of the area under the population size vs. time curve of LC20 exposure. Asterisks denote significant differences (* *P*<0.05; ** *P*<0.01; *** *P*<0.001) (ns = not significant; NA = not applicable).
(XLSX)

S6 Table Between treatment comparison of the maximum population size of LC20 exposure. Asterisks denote significant differences (* *P*<0.05; **P<0.01; *** *P*<0.001) (ns = not significant; NA = not applicable).
(XLSX)

S7 Table Between treatment comparison of the end population size of LC20 exposure. Asterisks denote significant differences (* *P*<0.05; ** *P*<0.01; *** *P*<0.001) (ns = not significant; NA = not applicable).
(XLSX)

S8 Table Between treatment comparison of the area under the population size vs. time curve of LC5 exposure. Asterisks denote significant differences (* *P*<0.05; ** *P*<0.01; *** *P*<0.001) (ns = not significant; NA = not applicable).
(XLSX)

S9 Table Between treatment comparison of the end population size of LC5 exposure. Asterisks denote significant differences (* *P*<0.05; ** *P*<0.01; *** *P*<0.001). (ns = not significant; NA = not applicable).
(XLSX)

S10 Table Observed mortality after 24 h of exposure to ZnCd mixtures compared between treatments and with predictions from the CA and IA model. Asterisks denote significant differences (* P<0.05; ** P<0.01; ***P<0.001) (ns = not significant; NA = not applicable).
(XLSX)

S11 Table Observed mortality after 48 h of exposure to ZnCd mixtures compared between treatments and with predictions from the CA and IA model. Asterisks denote significant differences (* *P*<0.05; ** *P*<0.01; *** *P*<0.001) (ns = not significant; NA = not applicable).
(XLSX)

S12 Table Between treatment comparison of the area under the population size vs. time curve of ZnCd mixture exposures. Asterisks denote significant differences (* P<0.05; ** P<0.01; *** P<0.001) (ns = not significant; NA = not applicable).
(XLSX)

S13 Table Between treatment comparison of the end population size of ZnCd mixture exposures. Asterisks denote significant differences (* *P*<0.05; ** *P*<0.01; *** *P*<0.001) (ns = not significant; NA = not applicable).
(XLSX)

S1 Fig Pictures taken at day 5 of 1/8 of the population of nematodes exposed to control (left) and LC20 of Cu (right) (magnification 6x). (TIF)
