# Supplementary figures and images for: The effect of metal mixture composition on toxicity to C. elegans at individual and population levels
Source: PLoS One. 2019 Jun 25;14(6):e0218929. doi: 10.1371/journal.pone.0218929 (PMC6592602; doi:10.1371/journal.pone.0218929)

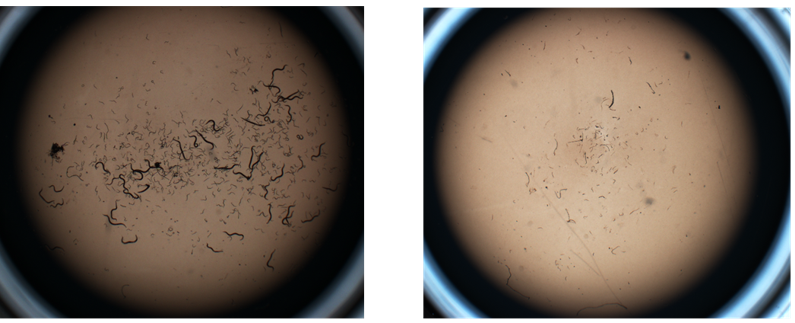

Supplement: S1 Fig — (TIF) [file pone.0218929.s014.tif]
